# *EPS8* Inhibition Increases Cisplatin Sensitivity in Lung Cancer Cells

**DOI:** 10.1371/journal.pone.0082220

**Published:** 2013-12-19

**Authors:** Lidija K. Gorsic, Amy L. Stark, Heather E. Wheeler, Shan S. Wong, Hae K. Im, M. Eileen Dolan

**Affiliations:** 1 Section of Hematology/Oncology, Department of Medicine, University of Chicago, Chicago, Illinois, United States of America; 2 Department of Health Studies, University of Chicago, Chicago, Illinois, United States of America; Univesity of Texas Southwestern Medical Center at Dallas, United States of America

## Abstract

Cisplatin, a commonly used chemotherapeutic, is associated with ototoxicity, renal toxicity and neurotoxicity, thus identifying means to increase the therapeutic index of cisplatin may allow for improved outcomes. A SNP (rs4343077) within *EPS8*, discovered through a genome wide association study of cisplatin-induced cytotoxicity and apoptosis in lymphoblastoid cell lines (LCLs), provided impetus to further study this gene. The purpose of this work was to evaluate the role of *EPS8* in cellular susceptibility to cisplatin in cancerous and non-cancerous cells. We used *EPS8* RNA interference to determine the effect of decreased *EPS8* expression on LCL and A549 lung cancer cell sensitivity to cisplatin. *EPS8* knockdown in LCLs resulted in a 7.9% increase in cisplatin-induced survival (*P* = 1.98×10^−7^) and an 8.7% decrease in apoptosis (*P* = 0.004) compared to control. In contrast, reduced *EPS8* expression in lung cancer cells resulted in a 20.6% decrease in cisplatin-induced survival (*P* = 5.08×10^−5^). We then investigated an *EPS8* inhibitor, mithramycin A, as a potential agent to increase the therapeutic index of cisplatin. Mithramycin A decreased *EPS8* expression in LCLs resulting in decreased cellular sensitivity to cisplatin as evidenced by lower caspase 3/7 activation following cisplatin treatment (42.7%±6.8% relative to control *P* = 0.0002). In 5 non-small-cell lung carcinoma (NSCLC) cell lines, mithramycin A also resulted in decreased *EPS8* expression. Adding mithramycin to 4 NSCLC cell lines and a bladder cancer cell line, resulted in increased sensitivity to cisplatin that was significantly more pronounced in tumor cell lines than in LCL lines (p<0.0001). An EGFR mutant NSCLC cell line (H1975) showed no significant change in sensitivity to cisplatin with the addition of mithramycin treatment. Therefore, an inhibitor of *EPS8*, such as mithramycin A, could improve cisplatin treatment by increasing sensitivity of tumor relative to normal cells.

## Introduction

Cisplatin is a platinum agent used for the treatment of head and neck, ovarian, cervical, testicular, and lung cancers; however severe toxicities and intrinsic/acquired resistance interfere with its efficacy [1]. Understanding genetic and molecular mechanisms by which this chemotherapeutic agent causes toxic side effects would be of great benefit to patients. In particular, identification of genes whose expression contributes to toxicity would allow for the development of chemotherapy to circumvent toxic effects.

Our lab has developed a preclinical pharmacogenomic model utilizing lymphoblastoid cell lines (LCLs) to identify genetic variants associated with susceptibility to chemotherapeutics to complement and enhance clinical pharmacogenomic studies [2]–[5]. Importantly, variants identified in the cell-based approach have been shown to be associated with response in ovarian cancer [6], lung cancer [7], head and neck cancer [8], and paclitaxel-induced peripheral neuropathy in breast cancer patients [9], providing confidence in the cell-based model for identifying clinically relevant variants.

For most LCL studies, drug-induced cell growth inhibition was the pharmacologic phenotype measured, however this is a broad phenotype that includes cellular processes leading to necrosis, cell death through apoptotic and non-apoptotic pathways, cell cycle arrest, and damaged cells undergoing DNA repair [10]. Apoptosis, a more specific phenotype, might shed light on relevant single nucleotide polymorphisms (SNPs) associated with cisplatin response in patients, since cisplatin is known to cause cell death through an apoptotic pathway [11]. Therefore, in a previous study we treated HapMap LCLs with cisplatin and measured caspase 3/7 activation as well as cell growth inhibition [12]. A GWAS revealed 2449 SNPs and 1629 SNPs suggestively associated with cisplatin-induced apoptosis and cytotoxicity (*P*<0.001), respectively, with 19 overlapping SNPs [12]. One of the common SNPs, rs4343077, in which the minor allele had lower cisplatin induced apoptosis (*P* = 0.0007) and higher survivial (*P* = 0.0007) is also an expression quantitative trait locus (eQTL) associated with the baseline gene expression levels of 28 genes at *P*≤10^−4^ [12]. This SNP is in an intron of epidermal growth factor receptor pathway substrate 8 (*EPS8*).

Interestingly, *EPS8* has been specifically linked to cisplatin- and paclitaxel-induced drug response, where cervical cancer cells became more sensitive to drug treatment following *EPS8* knockdown [13]. Recently, *EPS8* was also found to be overexpressed in human malignant gliomas and promoted their cellular growth [14]. Studies have reported increased expression of *EPS8* in other various human tumors including ovarian, colorectal, lung, pituitary and oral cancers [15]. As a result, *EPS8* attenuation has been shown to affect cell migration and cellular proliferation in cancer cells [15].

Due to the importance of *EPS8* in response to cisplatin in tumor cells [13] and our identification of a SNP within *EPS8* (rs4343077) associated with both cisplatin cytotoxicity and apoptosis [12], we further evaluated the relevance of *EPS8* in sensitivity to cisplatin. To this end, we used siRNA against *EPS8* and a known *EPS8* inhibitor, mithramycin. Downregulation using siRNA and/or inhibition of *EPS8* by mithramycin resulted in decreased greater cell growth inhibition in non-EGFR mutant lung cancer cells and a bladder cell line following cisplatin treatment. Our study identifies the importance of *EPS8* in cisplatin-induced cytotoxicity.

## Materials and Methods

### Cell lines

Nine LCLs (GM6991, GM7348, GM10838, GM11994, GM12239, GM10859, GM11830, GM11840, GM12156) derived from individuals of Northern and Western European ancestry (HapMap CEU) were maintained in RPMI 1640 media containing 15% fetal bovine serum (Hyclone, Logan, Utah, USA) and 20 mM L-glutamine. Cell lines were diluted 3 times a week to a concentration of 350,000 cells/ml. A549, NCI-H1437, NCI-H1563 and NCI-H1975 (human non-small-cell lung carcinoma cell lines) were maintained in RPMI 1640 containing 10% fetal bovine serum. NCI-H2126 (human non-small-cell lung carcinoma cell line) was maintained in DMEM:F12 containing 0.005 mg/ml insulin, 0.01 mg/ml transferrin, 30 nM sodium selenite, 10 nM hydrocortisone, 10 nM beta-estradiol, extra 2 mM L-glutamine and 5% fetal bovine serum (medium suggested by ATCC). HTB9 (urinary bladder grade II carcinoma cell line) was maintained in RPMI 1640 and 10% fetal bovine serum. All cell lines were stored in a 37°C incubator with 5% CO_2_. Cancer cells, medium and components were purchased from ATCC (Manassas, Virginia, USA), Cellgro (Herndon, Virginia, USA) or Sigma-Aldrich Co. (St. Louis, Missouri, USA).

### Drugs

Cisplatin and mithramycin A were purchased from Sigma-Aldrich Co. Dimethyl sulfoxide was used to dilute cisplatin to a 20 mM stock, whereas mithramycin was diluted to a stock concentration of 0.06 µM using phosphate buffered saline.

### Correlation between *EPS8* and phenotypes

Genome-wide gene expression data were generated in our lab with Affymetrix GeneChip Human Exon Array 1.0 ST Array [16] and all raw exon array data have been deposited into Gene Expression Omnibus (accession no. GSE7761). *EPS8* gene expression levels were correlated to 5 µM cisplatin induced cytotoxicity and apoptosis [12] in CEU LCLs (n = 77). Linear regression analyses between *EPS8* levels and each phenotype were performed using GraphPad Prism 4.

### RNA interference

Knockdown experiments were conducted to demonstrate the effects of lower *EPS8* levels on cisplatin-induced cytotoxicity and apoptosis. Using Lonza Cell Line 96-well Nucleofector Kit SF (Lonza Inc, Basel, Switzerland), LCLs and A549 were nucleofected 24 hrs after being seeded at 5.5×10^5^ cells/ml and 4.0×10^5^ cells/ml, respectively. Cells were centrifuged at 90× g for 10 minutes at room temperature and resuspended at a concentration of 1×10^6^ cells/20 µl in SF/supplement solution and 2 µM final concentration of AllStars Negative Control siRNA labeled with AlexaFluor488 (Qiagen Inc., Valencia, CA, USA) or a pool of Hs_EPS8 (SI00380737, SI03109302, SI00380751, SI00380744) FlexiTube siRNA (Qiagen). Program DN-100 was used for LCL nucleofection and CM-130 for A549. Cells were given 10 minutes rest prior to the addition of RPMI media, then plated for cytotoxicity or apoptosis and incubated overnight.

### Cytotoxicity following siRNA or treatment with mithramycin

An alamarBlue cellular growth inhibition assay was used to measure cytotoxic effects of cisplatin and mithramycin [17]. For *EPS8* siRNA experiments, LCLs (GM6991, GM7348, GM10838, GM11994 and GM12239) were treated with 5 µM cisplatin 5 hrs post nucleofection, while A549 was treated with 5 µM cisplatin 24 hrs after nucleofection. Following drug treatment cells were incubated for 24 hrs. AlamarBlue was then added and plates were incubated for an additional 24 hrs before being read at wavelengths of 570 and 600 nm using the Synergy HT (Biotek, Winooski, VT) and percent survival was calculated [12]. To observe cytotoxic response with *EPS8* knockdown through mithramycin, cells (GM10859, GM11830, GM11840, GM12156, A549, H1437, H1563, H1975, H2126 and HTB9) were plated and treated with either mithramycin alone (0 and 0.01 µM), cisplatin alone (0, 1, 2.5, 5, 10 20, 25 and 50 µM) or cisplatin at various concentrations combined with 0.01 µM of mithramycin. Mithramycin treatment occurred immediately after plating. Cells were then treated with cisplatin 6 hrs post mithramycin addition and 10% of the total well volume of alamarBlue was added 24 hrs after cisplatin treatment. Following an additional 24 hr incubation period, plates were read as stated above. All experiments for percent survival measurements were plated in triplicate with a minimum of two separate experiments.

### Apoptosis assay

Cisplatin- and mithramycin-induced apoptosis were measured using Caspase-Glo 3/7 reagent from Promega Corporation (Madison, WI) as previously described [12]. For *EPS8* siRNA experiments, plated cells were treated with 5 µM cisplatin 5 hrs post nucleofection and caspase 3/7 was measured 24 hrs after cisplatin treatment. To evaluate *EPS8* gene expression following mithramycin exposure, cells were plated and immediately treated with mithramycin (0 or 0.01 µM), then treated with 5 µM cisplatin 20 hrs after mithramycin addition. Caspase 3/7 activity was measured 24 hrs after cisplatin and calculated as relative to control (no drug addition). Results for apoptosis measurements represent experiments plated in triplicate with a minimum of two independent repeats.

### Quantifying knockdown of *EPS8*

Cells were pelleted at 5, 29, and 53 hrs after nucleofection with *EPS8* siRNA and scrambled control, as well as 6 and 20 hrs following mithramycin treatment. RNA was extracted using the RNeasy Plus Mini kit and QIAcube (Qiagen) following the manufacturer's protocol. mRNA was then reverse transcribed to cDNA yielding final concentrations of 25 or 50 ng/µL using the High Capacity Reverse Transcription kit (Applied Biosystems, Life Technologies, Grand Island, NY). The cDNA was used to perform qRT-PCR to confirm the knockdown of the *EPS8* [18]. Applied Biosystem TaqMan primer was used to quantify mRNA expression of *EPS8* (Hs00610286_m1).

### Mixed effects model

The effect of *EPS8* knockdown on cisplatin-induced apoptosis and growth inhibition was modeled using the following mixed effects model:
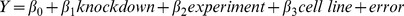



Experiment and cell lines were random effects allowing for experiment specific and cell line specific intercepts and knockdown status was considered a fixed effect. Y represents apoptosis or growth inhibition. P values for knockdown effect were calculated using the likelihood ratio test with models fit with REML set to false. Goodness of fit of the models was assessed examining the residuals' distributions. R Statistical software (R Development Core Team http://www.R-project.org/) and the *lme4* package (R package version 0.999375-42 http://CRAN.R-project.org/package=lme4) were used for analysis.

### Interaction of mithramycin ± cisplatin

To test the interaction effect of cell type (tumor vs LCL) and mithramycin treatment on the cisplatin dose response curve we fit a mixed effects. The final model was:
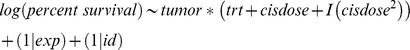
where the natural logarithm of percent survival was the outcome and tumor (status vs LCL status) and mithramycin treatment (trt) were fixed effects with interaction term; exp indicated one of the two biological replicates and was fitted as random effect; cisplatin dose and its square were used to model the dose response curve allowing to be different for tumor and LCL lines; cell line id was added as a random effect to account for within cell line correlation. When fitting the model the outcomes at zero concentration of both drugs (rows where survival outcome = 1 because of the way percent survival was computed) were excluded. Log transformation was used to improve model fit. The coefficient of the interaction term tumor∶trt can be interpreted as the average shift of the dose response curve when mithrmycin was added to tumor lines relative to LCL lines.

## Results

### Successful knockdown of *EPS8* through RNA interference

**Figure 1:**
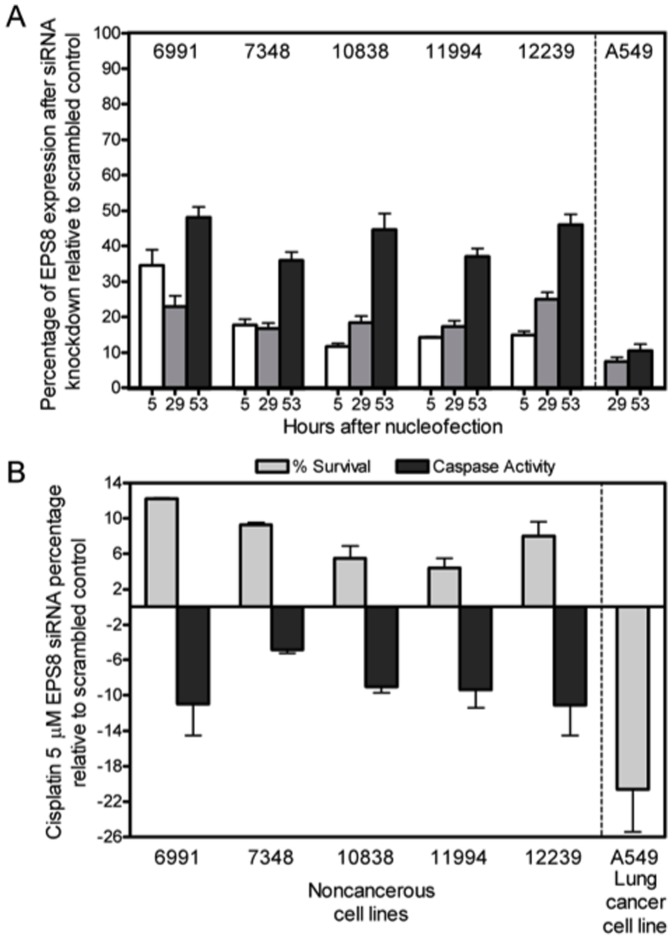
Percent of *EPS8* expression compared to scrambled control with standard error of the mean is shown for 5 LCLs tested at 5, 29 and 53 hrs after nucleofection and cell line A549 at 29 and 53 hrs after nucleofection (A). Values include 2 independent experiments with qRT-PCR run in duplicate. Cell line changes in percent survival (*P* = 1.98×10^−7^) and caspase 3/7 activity (*P* = 0.004) for all LCLs and A549 (*P* = 5.08×10^−5^) at 5 µM cisplatin due to *EPS8* knockdown are shown with standard error of the mean using 6 replicates from 2 independent experiments (B).

Five CEU LCLs and the A549 lung cancer cell line were utilized for studies involving *EPS8* knockdown. Cells were nucleofected with either a scrambled control or siRNA of *EPS8*. Comparing to the scrambled control, *EPS8* knockdown was shown to be successful across all 6 cell lines (Fig. 1A). The average percentages of *EPS8* across all LCLs at the 5, 29, and 53 h time points were 17.2 (±7.5), 19.0 (±4.3), and 40.6% (±7.3), respectively. A549 achieved *EPS8* knockdown to 7.4% and 10.5% compared to scrambled control at 29 and 53 hours, respectively.

### Phenotypic changes with *EPS8* siRNA

After confirming knockdown of *EPS8*, we evaluated the changes in sensitivity to cisplatin as measured by percent survival and caspase 3/7 activation. The correlation between *EPS8* expression and cisplatin-induced cytotoxicity indicated lower levels of *EPS8* expression significantly correlated to greater percent survival at 5 µM cisplatin (*P* = 0.047); however cisplatin induced apoptosis did not reach significance (*P* = 0.499) (Fig. S1). Using a mixed effects model combining all cell lines, *EPS8* RNA interference showed an increased survival of cisplatin-treated LCLs by an average of 7.9% (*P* = 1.98×10^−7^) and decreased apoptosis by an average of 8.7% (*P* = 0.004) (Fig. 1B). These results reveal that lower levels of *EPS8* in LCLs decrease cellular sensitivity to cisplatin as evidenced by increased cell survival and reduced apoptotic activity when treated with cisplatin. Thus, decreased *EPS8* in LCLs confers resistance to cisplatin toxicity. In contrast, *EPS8* downregulation in A549 caused greater sensitivity to cisplatin with a 20.6% decrease in percent survival (*P* = 5.08×10^−5^) (Fig. 1B).

### Mithramycin reduces expression of *EPS8* in cancer lines and LCLs

**Figure 2:**
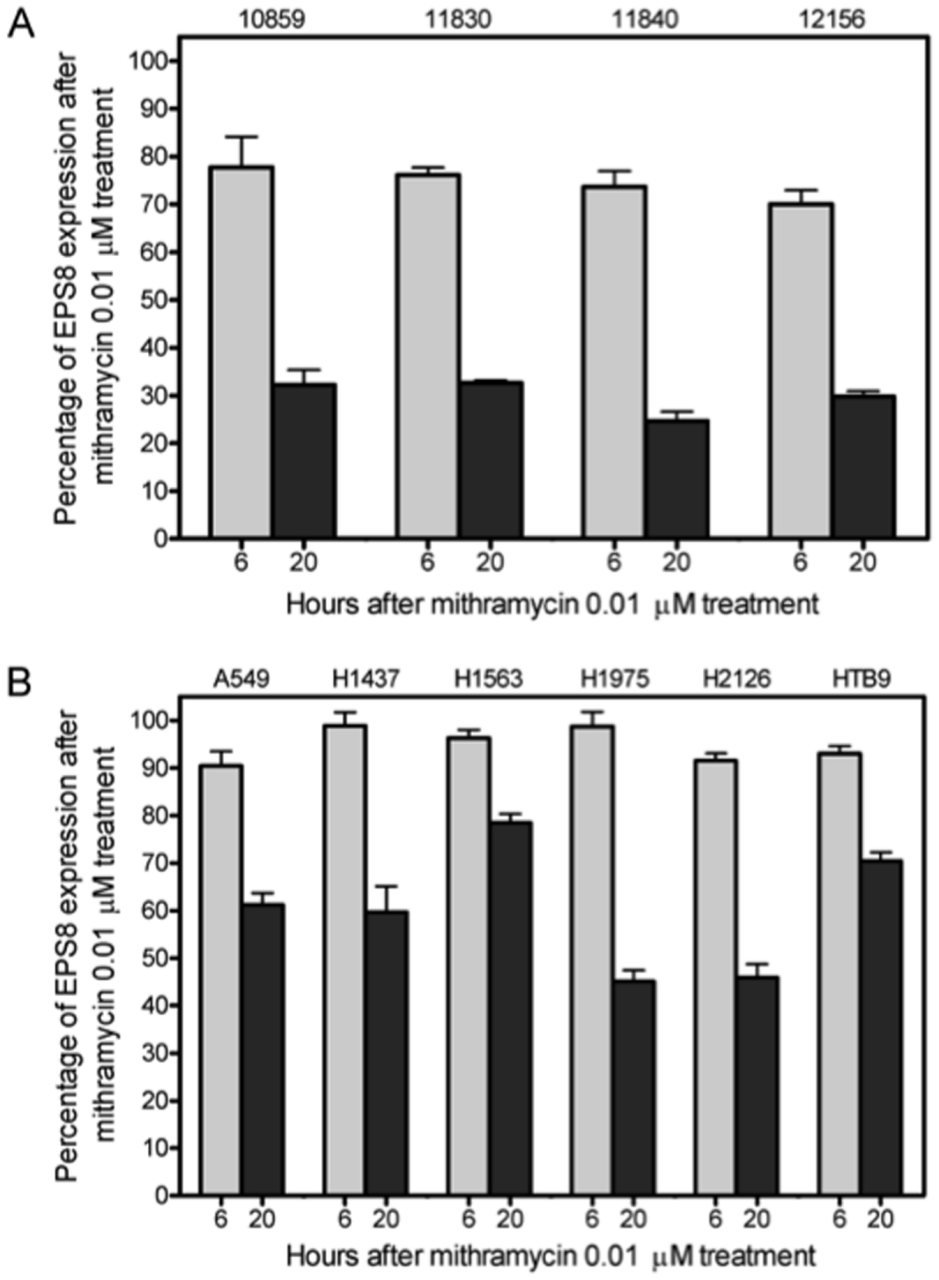
Percentage of *EPS8* expression following treatment with 0.01 µM mithramycin compared to no drug control at 6 and 20 hrs after exposure for each LCL tested (A) and cancerous cell line (B). Percent values shown include two independent experiments with qRT-PCR run in duplicate for each experiment with standard error of the mean.

Levels of *EPS8* expression were measured 6 and 20 hrs following treatment with mithramycin (0.01 µM). Levels of *EPS8* expression decreased across all 4 LCLs tested, with an average of 74.4% (±3.4) at 6 hrs and 29.8% (±3.7) at 20 hrs relative to control following mithramycin exposure (Fig. 2A). Mithramycin also decreased *EPS8* expression levels in 5 NSCLC cell lines and bladder cancer cell line to an average of 95.7% (±3.4) at 6 hrs and 59.9% (±14.7) at 20 hrs of exposure, compared to no drug treatment control (Fig. 2B). These measurements confirm that the treatment of mithramycin (0.01 µM) results in lower expression of *EPS8* in cancerous lung and bladder cells as well as in noncancerous LCLs.

### Mithramycin decreases sensitivity of LCLs to cisplatin

**Figure 3:**
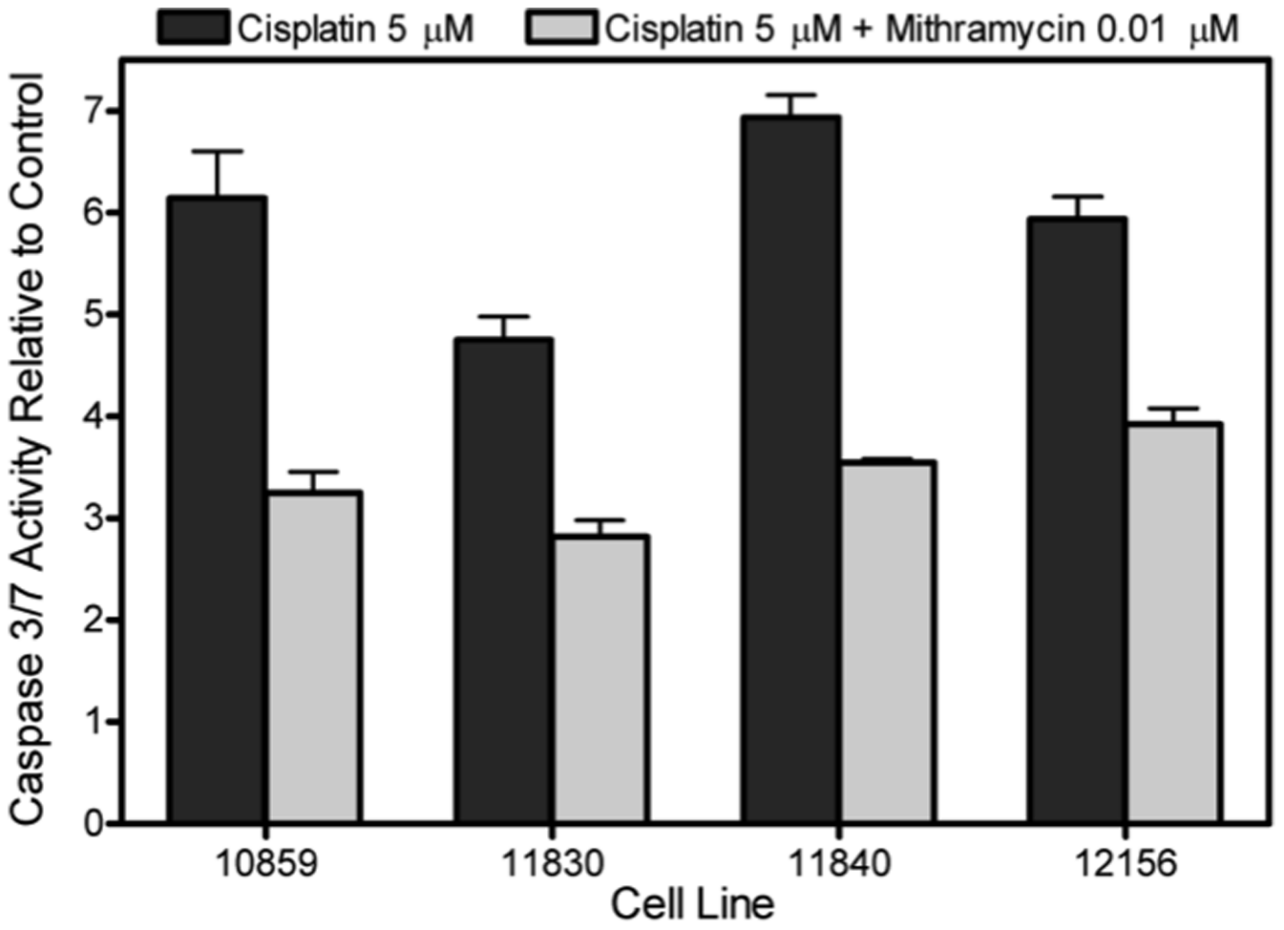
LCL caspase 3/7 activity for 5 µM cisplatin treatment alone compared to cisplatin plus 0.01 µM mithramycin. Each LCL experienced lower cisplatin-induced apoptotic activity with the added mithramycin relative to a no drug treatment control. Mithramycin treated 10859, 11830, 11840, and 12156 resulted in a 47.1, 40.8, 48.9, and 34.0% decrease from cisplatin alone, respectively. Data represents two separate experiments, each done in triplicate with standard error of the mean.

We then determined the effect of mithramycin on sensitivity of LCLs to cisplatin-induced caspase 3/7 activation. The average apoptosis levels across the 4 LCLs following cisplatin alone was 5.94 (±0.9) relative to control, in comparison to cisplatin plus mithramycin which decreased the average caspase 3/7 levels to 3.38 (±0.5) (Fig. 3). Caspase 3/7 activity after mithramycin alone (0.01 µM) resulted in an average of 3.41 (±0.4) across the 4 LCLs. Even though mithramycin and cisplatin separately induce apoptosis, there was an average decrease of 42.7% (±6.8 *P* = 0.0002) in caspase 3/7 activation by combining mithramycin with cisplatin compared to cisplatin alone, suggesting a protective effect of mithramycin in terms of apoptosis. Lung and bladder cancer cells were also tested for apoptosis with cisplatin (5 µM) in the presence and absence of mithramycin; however caspase 3/7 activation levels for these cells were not above baseline.

### Mithramycin enhances sensitivity of tumor cells to cisplatin

**Figure 4:**
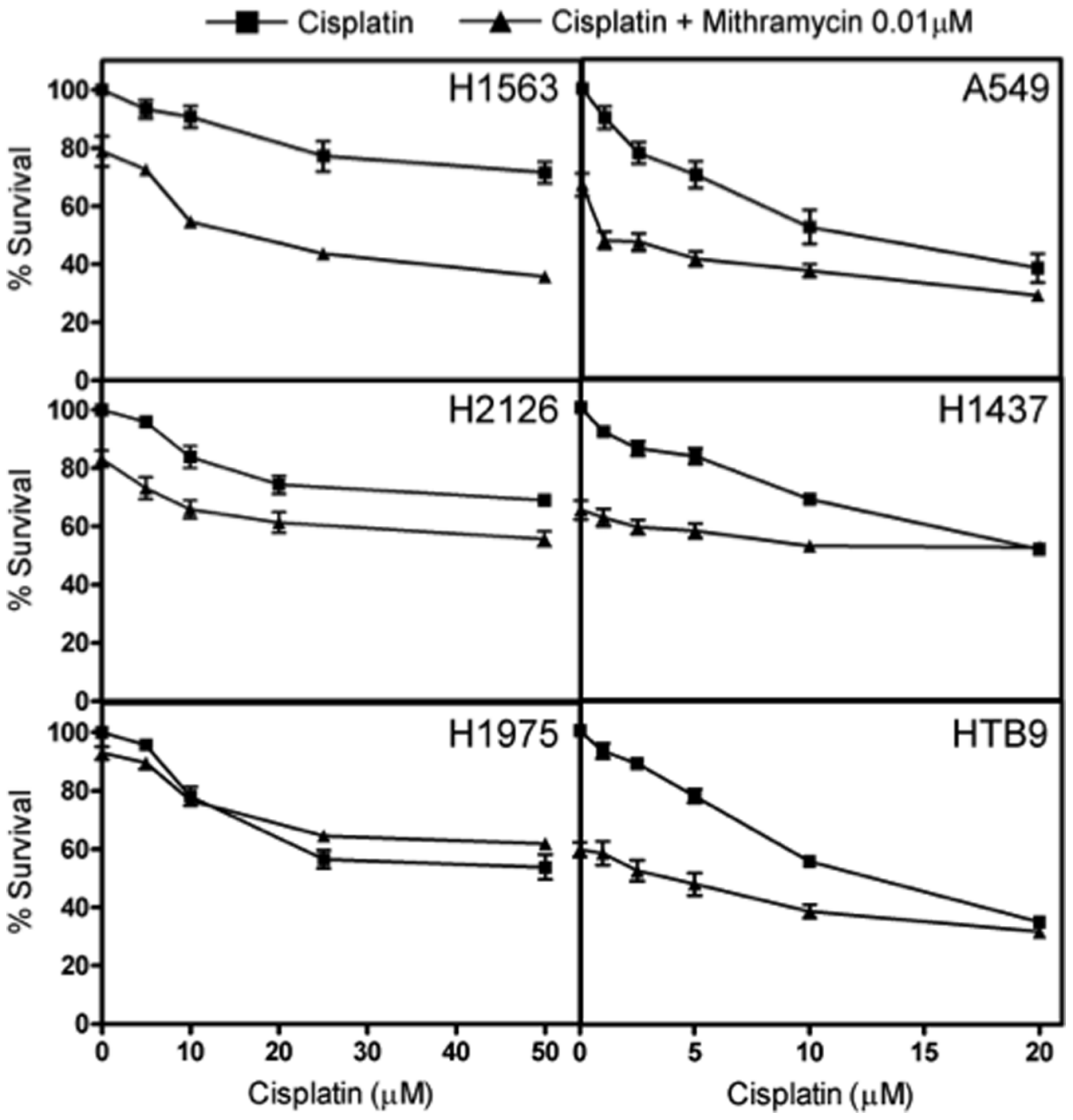
NSCLC cell lines and bladder cell line, HTB9, are shown for various concentrations of cisplatin with the presence and absence of mithramycin. Square shape represents cisplatin concentrations alone, while the triangle represents cisplatin with the addition of mithramycin (0.01 µM). Curves represent two independent experiments done in triplicate with standard error of the mean.

In addition to testing apoptosis levels with cisplatin and mithramycin in LCLs and cancer cells, we also measured cell growth inhibition. Five NSCLC cell lines with diverse mutation statuses were chosen for study; the effect of mithramycin alone varies for these cancer cell lines ranging from 59.7 to 92.9% cell growth inhibition (Table 1). Sensitivity to cisplatin was found to increase with mithramycin treatment in 4 molecularly distinct NSCLC cells (Fig. 4). H1975 with an EGFR mutation, experienced no significant change. Since cisplatin is also used for the treatment of bladder cancer, we also chose to measure cisplatin and mithramycin effects in a bladder tumor line to see whether the effect would emulate NSCLC results; we observed 59.6% survival with mithramycin treatment alone (Fig. 4).

**Table 1:** Effect of mithramycin on sensitivity of molecularly distinct non-small-cell lung carcinoma cell lines.

NSCLC Cell Line	KRAS	TP53	EGFR	STK11	Mithramycin (0.01 µM)
A549	mt	+	+	+	59.7%
H1437	+	mt	+	+	65.0%
H1563	+	+	+	+	78.9%
H1975	+	mt	mt	+	92.9%
H2126	+	mt	+	mt	81.2%

+ : Wild-type mt : Mutant.

*Mutation status of each cell line was provided from ATCC (Manassas, Virginia).

However, when examining the effect of mithramycin on sensitivity of LCLs to cisplatin, we did not observe the same degree of enhanced cell growth inhibition. Mithramycin alone across 4 LCLs caused an average cell growth inhibition of 63.1% (±12.8) at 0.01 µM (Fig. S2). The dose response curve for LCLs was shifted downward on average about 17% when mithramycin was added. For tumor lines the dose response curve shifted downwards roughly 26% on average. The p-value of the interaction term was *P*<0.0001. This, along with apoptosis results, supports the notion that reduced expression of *EPS8* via mithramycin is not equally harmful to LCL survival as it is for cancerous cell lines. Mithramycin causes greater sensitivity in NSCLC cells without EGFR mutation and possibly other cancer tissue types as seen by our results in bladder tumor cell line, HTB9.

## Discussion

In this study, we evaluated *EPS8* as a potential target for combination therapy with cisplatin. *EPS8* was chosen based on previous preclinical GWAS results using multiple cellular phenotypes: cisplatin-induced cytotoxicity and cisplatin-induced apoptosis as measured by caspase 3/7 activation. The SNP (intronic to *EPS8*), rs4343077, was associated with cisplatin-induced cytotoxicity and apoptosis as well as baseline expression of 28 target genes. Upon knockdown of *EPS8* expression in 5 LCLs, we observed a significant decrease in cellular sensitivity to cisplatin as measured by cell growth inhibition and caspase 3/7 activation (*P* = 1.98×10^−7^ and *P* = 0.004, respectively) across LCLs. Literature evidence suggested *EPS8* knockdown in tumor cell lines increased cellular sensitivity to cisplatin [13], [15]. Our results agree with these findings, showing that *EPS8* downregulation sensitizes A549 lung cancer cells to cisplatin treatment. We extended our studies to other molecularly defined lung cell lines and a bladder cell line as well as LCLs. *EPS8* knockdown in LCLs following mithramycin treatment resulted in a decrease in apoptotic activity when mithramycin was combined with cisplatin compared to cisplatin alone. Although cell cytotoxicity measurements indicated a small increase in sensitivity of LCLs to cisplatin following mithramycin exposure, sensitivity of cancerous cell lines was significantly greater with mithramycin addition compared to LCLs. The exception was the NSCLC cell line H1975 with an EGFR mutation. This implies knockdown of *EPS8* through either siRNA or use of an inhibitor such as mithramycin may be a strategy to increase tumor sensitivity to cisplatin.

EPS8 is an oncoprotein contributing to malignant transformation in tumor cells [12], [19]. It is a substrate of epidermal growth factor receptor (EGFR) and participates in EGFR signaling through Rac, and trafficking through Rab5 [20]. Its tri-complex relationship with *SOS1* and *ABI1* has been found to be an essential component for lysophosphatidic acid-stimulated cell migration and Rac activation, which has been found to play an important role in ovarian cancer metastasis [21]. Levels of *EPS8* have also been implemented as a prognostic tool for patients during early stages of cervical cancer [13]. Patients having higher expression of *EPS8* tend to experience parametrial invasion, lymph node metastasis and a decrease in survival rate.

Initially, Yang et al. (2010), investigated mithramycin, an antibiotic from the *Streptomyces* species, as a potential inhibitor of *EPS8*. They determined that mithramycin reduces the mRNA and protein levels of *EPS8* in a human colorectal adenocarcinoma epithelial cell line and a human lung carcinoma epithelial cell line. Furthermore, *EPS8* downregulation, through mithramycin treatment, causes a significant decrease in cancer cell growth and migration ability [22].

Mithramycin has been an approved clinical anticancer drug since 1970 [23] and used in the United States for clinical treatment of Paget's disease, testicular carcinoma, and hypercalcemia in patients who experience malignancy-associated bone lesions [24]–[28]. However, its use in therapy treatment has diminished throughout the years due to its adverse effects and narrow therapeutic index [29]. Patients treated with mithramycin, for these diseases, have been seen to experience gastrointestinal, hepatic, renal and bone marrow toxicities, resulting in nausea, vomiting, and bleeding [30]. Despite its severe side effects, there has been a renewed interest in mithramycin now that understanding of its interactions at the molecular level is evolving [29].

Mithramycin has been found to interact with GC-rich DNA regions located at the minor groove of DNA [29], [31]–[33], which is currently thought to prevent transcription factor specificity protein 1 (Sp1) from binding to a variety of promoters of proto-oncogenes. However, Sp1 binding sites that are unrelated to a subset of proto-oncogenes seem to remain unaffected, such as promoter p21^cip1/waf1^ [29]. Therefore, mithramycin may not be specific to Sp1 inhibition and could potentially target an oncogene upstream of Sp1 interaction. It has been previously discovered that mithramycin also decreases expression of *c-myc*, *c-myb*, *c-src*, *c-met*, and FOXM1 [22], [34]; however, mithramycin increases levels of FOXO3A [34], a transcription factor which regulates the DNA damage response. There may be a possibility that these genes are interrelated in a pathway downstream of mithramycin's target.

For instance, *EPS8* has also been shown to upregulate FOXM1 [35], an important factor in the development and progression of certain cancers [36] which is known to directly bind with Sp1 [37], [38]. Sp1 and FOXM1 have been shown to transactivate promoters of *c-myc* synergistically [38]. Furthermore, *EPS8* attenuation has been found to decrease levels of *Src*, *Shc* and *FAK* (also known as *PTK2*), an intracellular tyrosine kinase participating in cell adhesion and motility [39]. FOXO3A may also be regulated by the presence or absence of *EPS8* through various pathways, either through PI3K/AKT/mTOR signaling [40] or via the Ras-Raf-MEK-ERK pathway [41]. Shiota et al. (2010) investigated cisplatin resistance and determined that cisplatin resistant cells became sensitized to treatment through the induction of FOXO3A by mithramycin [34]. A decrease in AKT signaling activates FOXO3A and induces apoptosis [36]; therefore, it is possible that a reduction in *EPS8* levels decreases AKT, triggering phosphorylation of FOXO3A and cancer cells to undergo apoptosis instead of continued proliferation.

Taking a collective look at the literature and our results it seems interesting that the lung cancer cell line which did not experience increased cell death with mithramycin (H1975) has an EGFR mutation. Lung tumor cell line H1975 has a point mutation in the activation loop causing a change from leucine to arginine (L858R) in exon 21 as well as a secondary point mutation, T790M, altering normal EGFR activity [42]. EGFR tyrosine inhibitors are typically used to sensitize EGFR mutated tumor types to platinum agents, however EGFR wild-type tumors rarely response to these inhibitors [43]. Potentially, the addition of a mithramycin regimen may be beneficial to patients with wild-type EGFR to become sensitized to platinum treatment through inhibition of *EPS8* and downstream targets which play a large role in tumor cell proliferation, adhesion and motility. A mithramycin dose large enough to inhibit certain oncogenes, yet low enough not to cause additional adverse effects, would allow the chemotherapeutic agent to more effectively cause cellular death of tumor cells.

In conclusion, our results validate *EPS8* involvement in cell response to cisplatin treatment. Although we tested mithramycin, there may be more specific inhibitors of *EPS8* that result in a greater differential between cancer cells and normal cells. Mithramycin's ability to decrease levels of *EPS8* caused less apoptotic activity in LCLs than with cisplatin treatment alone. Reduced expression of *EPS8* through treatment with mithramycin is less harmful to normal cells (as measured in LCLs) compared to cancer cells. Collectively, our data provides further confirmation of the role and importance of *EPS8* in cisplatin-induced toxicity and as a promising target for improving cisplatin therapy.

## Supporting Information

Figure S1 **Correlation between** ***EPS8*** **expression levels and CEU LCL log transformed percent survival (** ***P*** **= 0.047, r^2^ = 0.05, top) and caspase 3/7 activity (** ***P*** **= 0.499, r^2^ = 0.006, bottom).**
(TIFF)

Figure S2 **LCLs are shown at various concentrations of cisplatin with the presence and absence of mithramycin.** Square shape represents cisplatin concentrations alone, while the triangle represents cisplatin with the addition of mithramycin (0.01 µM). Curves represent two independent experiments done in triplicate with standard error of the mean.
(TIFF)
