# Correction: EPS8 Inhibition Increases Cisplatin Sensitivity in Lung Cancer Cells

**DOI:** 10.1371/journal.pone.0091411

**Published:** 2014-02-27

**Authors:** The PLOS ONE Staff

There is an error in the second to last sentence of the "Introduction" section. The correct sentence for this section is:

"Downregulation using siRNA and/or inhibition of EPS8 by mithramycin resulted in greater cell growth inhibition in non-EGFR mutant lung cancer cells and a bladder cell line following cisplatin treatment."
